# Supplementary material for: Identification of photoperiod-induced specific miRNAs in the adrenal glands of Sunite sheep (Ovis aries)
Source: Front Vet Sci. 2022 Jul 22;9:888207. doi: 10.3389/fvets.2022.888207 (PMC9354845; doi:10.3389/fvets.2022.888207)
Supplement: Supplementary file 1 [file Data_Sheet_1.ZIP › Supplementary file/miRDeep2 file.zip!/miRDeep2 file/Novel_Structure/SP42A1/Novel_645.pdf]

Provisional ID	:	NC_019473.2_19213
Score total	:	2.8
Score for star read(s)	:	-1.3
Score for read counts	:	0
Score for mfe	:	2.5
Score for randfold	:	1.6
Score for cons. seed	:	
Total read count	:	25
Mature read count	:	24
Loop read count	:	0
Star read count	:	1

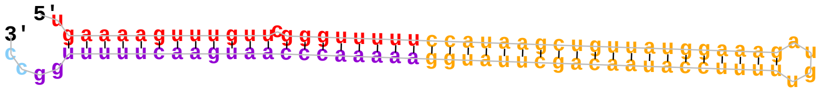

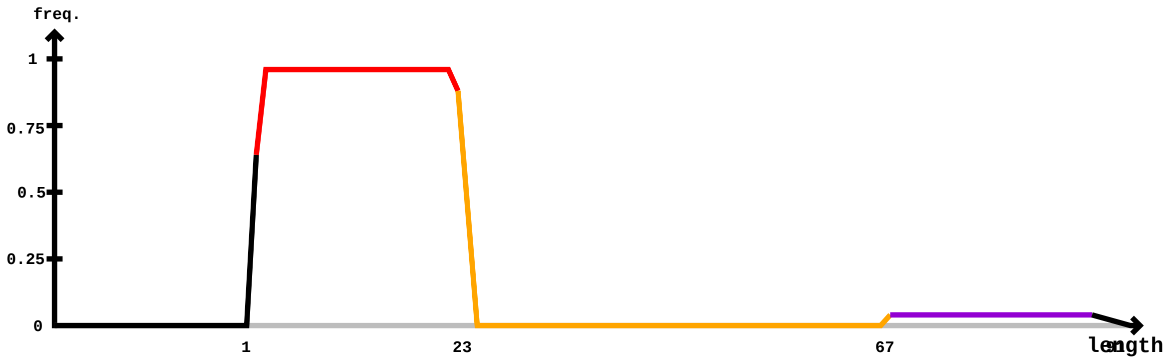


```
                      Mature                                                                       Star
5'- cccacagacacuggguuggugaaaaguuuguucggguuuuuccauaagcuguuauggaaagauguuuuuccauaacagcuuauggaaaaacccaaugaacuuuuuggccaa   -3'    obs
    cccacagacacuggguuggugaaaaguuuguucggguuuuuccauaagcuguuauggaaagauguuuuuccauaacagcuuauggaaaaacccaaugaacuuuuuggccaa          exp
    ((((.......))))(((((.((((((((((.(((((((((((((((((((((((((((((....)))))))))))))))))))))))))))))))))))))))..)))))    reads   mm   sample
    ...................ugaaaaguuuguucggguuuu.......................................................................    2       0    seq
    ...................ugaaaaguuuguucggguuuuu......................................................................    13      0    seq
    ...................ugaaaaguuuguucggguuuuuc.....................................................................    1       0    seq
    ....................gaaaaguuuguucggguuuuu......................................................................    8       0    seq
    .....................................................................................aaaaacccaaugaacuuuuugg....    1       0    seq
```
